# Supplementary material for: Content-rich biological network constructed by mining PubMed abstracts
Source: BMC Bioinformatics. 2004 Oct 8;5:147. doi: 10.1186/1471-2105-5-147 (PMC528731; doi:10.1186/1471-2105-5-147)
Supplement: Additional File 2 — The original results of the above study (non-essential files are deleted to keep the file size under the limit set by BMC bioinformatics). [file 1471-2105-5-147-S2.bz2 › chilibotAdditionalFile2/dip10/466ID10556181E10494/html/CCR4_CXCL12.html]

 


 **CCR4** and **CXCL12** 
  
Found 65 abstracts in PubMed, retrieved 10.  
 

 What does Google say? 
 PDF only 
| .edu only 

---

**Interactive relationship** (e.g. stimulation, inhibition, etc)

**Neutral relationship**- A simplified model of the binding interaction between stromal cell derived factor 1  [ **CXCL12** ]  chemokine and CXC chemokine receptor 4  [ **CCR4** ] .  Ref: 12678810 Protein Pept Lett, 2003
- Three chemokines in particular,  **CXCL12**  SDF 1  [ **CXCL12** ] , CCL17 TARC, and CCL22 MDC, recently have been shown to be strong and rapid activators of platelet aggregation and adhesion after their binding to platelet CXCR4 or  **CCR4** , when acting in combination with low levels of primary agonists.  Ref: 12851650 Microcirculation, 2003

**Non-interactive relationship** (e.g. studied together, co-existance, homology, etc.)

- We show that both CXC chemokine receptor 4  [ **CCR4** ]  CXCR4 and SDF 1  [ **CXCL12** ]  mRNA are expressed in several human glioblastoma multiforme tumor tissues and in two human glioblastoma cell lines, U87 MG and DBTRG 05MG.  Ref: 12702590 Cancer Res, 2003
- The Stromal Derived Factor 1  **CXCL12**  CXC Chemokine Receptor 4  [ **CCR4** ]  Biological Axis in Non Small Cell Lung Cancer Metastases.  Ref: 12626353 Am J Respir Crit Care Med, 2003
- In contrast to in vivo homing, both in vitro migration toward stromal derived factor SDF 1  [ **CXCL12** ]  and the average CXC chemokine receptor 4  [ **CCR4** ]  CXCR4 expression were lower in 5 FU treated cells.  Ref: 12829029 Exp Hematol, 2003
- Here we identify the CXC chemokine receptor 4  [ **CCR4** ]  CXCR4 in interneuron precursors migrating from the basal forebrain to the neocortex and demonstrate that stromal cell derived factor 1  [ **CXCL12** ]  SDF 1  [ **CXCL12** ]  is a potent chemoattractant for isolated striatal precursors.  Ref: 12832536 J Neurosci, 2003
